# Effect of antibiotic treatment on *Oxalobacter formigenes* colonization of the gut microbiome and urinary oxalate excretion

**DOI:** 10.1038/s41598-021-95992-7

**Published:** 2021-08-12

**Authors:** Lama Nazzal, Fritz Francois, Nora Henderson, Menghan Liu, Huilin Li, Hyunwook Koh, Chan Wang, Zhan Gao, Guillermo Perez Perez, John R. Asplin, David S Goldfarb, Martin J Blaser

**Affiliations:** 1 New York University Langone Health, New York University, New York, USA; 2 Vilcek Institute of Graduate Biomedical Sciences, New York University Langone Health, New York, USA; 3 Division of Biostatistics, Department of Population Health, New York University Langone Health, New York University, New York, USA; 4 Department of Applied Mathematics and Statistics, The State University of New York, Korea, Incheon, 21985 South Korea; 5 Center for Advanced Biotechnology and Medicine, Rutgers University, 679 Hoes Lane West, Piscataway, NJ 08854-8021 USA; 6 Litholink Corp, Chicago, USA

**Keywords:** Renal calculi, Bacterial physiology, Symbiosis, Antibiotics

## Abstract

The incidence of kidney stones is increasing in the US population. Oxalate, a major factor for stone formation, is degraded by gut bacteria reducing its intestinal absorption. Intestinal *O. formigenes* colonization has been associated with a lower risk for recurrent kidney stones in humans. In the current study, we used a clinical trial of the eradication of *Helicobacter pylor*i to assess the effects of an antibiotic course on *O. formigenes* colonization, urine electrolytes, and the composition of the intestinal microbiome. Of 69 healthy adult subjects recruited, 19 received antibiotics for *H. pylori* eradication, while 46 were followed as controls. Serial fecal samples were examined for *O. formigenes* presence and microbiota characteristics. Urine, collected serially fasting and following a standard meal, was tested for oxalate and electrolyte concentrations. *O. formigenes* prevalence was 50%. Colonization was significantly and persistently suppressed in antibiotic-exposed subjects but remained stable in controls. Urinary pH increased after antibiotics, but urinary oxalate did not differ between the control and treatment groups. In subjects not on antibiotics, the *O. formigenes*-positive samples had higher alpha-diversity and significantly differed in Beta-diversity from the *O. formigenes*-negative samples. Specific taxa varied in abundance in relation to urinary oxalate levels. These studies identified significant antibiotic effects on *O. formigenes* colonization and urinary electrolytes and showed that overall microbiome structure differed in subjects according to *O. formigenes* presence. Identifying a consortium of bacterial taxa associated with urinary oxalate may provide clues for the primary prevention of kidney stones in healthy adults.

Nephrolithiasis (kidney stones) affect up to 9 percent of the US population^1^, affecting both males and females, with incidence increasing in children and adults^2,3^. Most stones are composed of calcium oxalate (CaOx)^4,5^. Oxalate is absorbed in the gut from the diet and also produced endogenously as an end-product of amino acid metabolism^6,7^. Human intestinal bacteria that degrade oxalate and contribute to its metabolism are considered the o*xalobiome*^8^. Increasing evidence suggests gut microbiota roles in nephrolithiasis pathogenesis^9–11^.

Prior antibiotic use has been associated with kidney stone development months or even years later^12,13^. Although multiple bacterial taxa are able to degrade oxalate in the gut^8,14^, *Oxalobacter formigenes* is the only commensal known to use oxalate as its sole energy and carbon source and may be the only specialist oxalate degrader in humans^15^. The role in overall oxalate metabolism of other high abundance microbiota that have the capability to degrade oxalate but not use it as their primary energy source remains unknown. In our recent work, we showed that in healthy subjects, *O. formigenes* is the major reservoir of oxalate metabolizing genes at the transcriptional level, greater than all other organisms combined^16^. In rodent models, colonic colonization with *O. formigenes* significantly reduced urinary oxalate excretion^17–19^. Intestinal *O. formigenes* colonization has been associated with lower risk for recurrent kidney stones in humans^20^.

Epidemiologic studies provide evidence that *O. formigenes* prevalence in developed countries such as the USA is lower than in developing countries^21,22^. The prevalence of *O. formigenes* colonization is very high (> 80%) in uncontacted Amerindians^21^, and the hunter-gatherer Hadza people^22^, and was estimated at 60% in India^23^. Prevalence has been found to be ~ 30% in US and UK populations^24^. *O. formigenes* is susceptible to commonly used antibiotics^25^; an antibiotic course for *H. pylori* eradication led to loss of colonization for at least six months post-treatment^26^, but post-treatment examination of urinary analytes and microbiome were not done.

In the current study, we used a study (ESSAY) of the eradication of *Helicobacter pylori* to assess the effect of an antibiotic course on *O. formigenes* colonization and on urine electrolytes, including oxalate excretion (Fig. 1A). We also asked whether microbiome structure and composition differed over time with respect to both *O. formigenes* status and antibiotic exposure.

**Figure 1:**
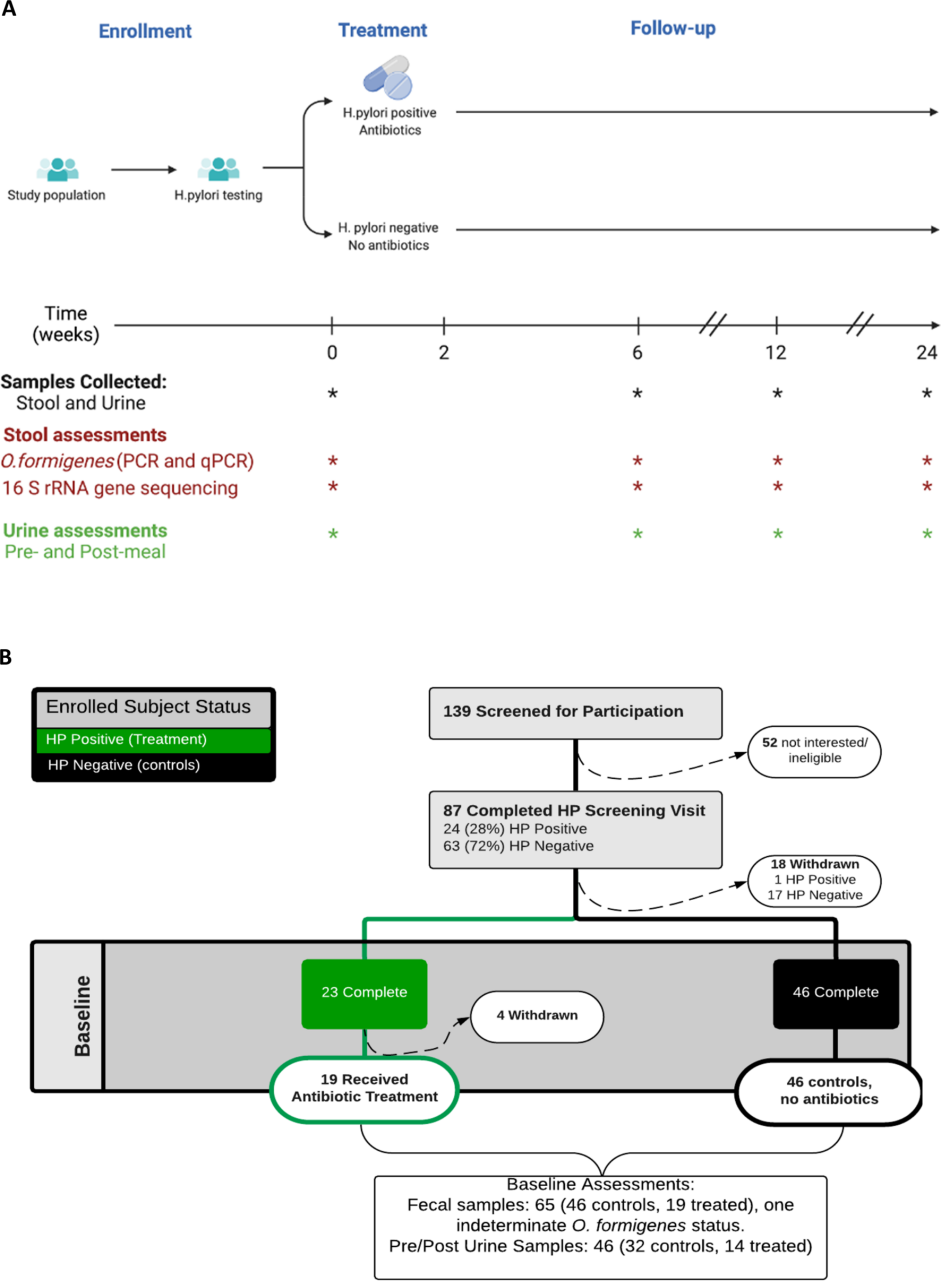
Study design and enrollment. Panel (**A**) Study design and assessments. From all enrolled subjects, baseline samples (stool and urine) were obtained at time 0. *H. pylori-*positive subjects received antibiotics for 2 weeks while *H. pylori*-negative subjects were followed as controls. Follow-up collections were performed at weeks 6, 12, and 24. Urine was collected after fasting (Pre-meal), and for 3 h following a standard meal (Post-meal). Stool samples were tested for O. formigenes using PCR and qPCR and underwent 16S rRNA gene sequencing. Urine was tested for multiple analytes. Panel created by BioRender software (www.biorender.com. Panel (**B**) Enrollment. Of 139 subjects screened for participation, 87 completed the screening, and 69 subjects underwent baseline assessment. 19 *H.pylori*-positive subjects were treated with antibiotics ,whereas 46 *H.pylori*-negative subjects served as controls. Panel created by Lucidchart software (www.lucidchart.com).

## Results

### Recruitment

Of 69 eligible participants who returned for the baseline visit, 23 (33%) tested positive for *H. pylori* while 46 tested negative; 65 of the 69 participants also provided fecal samples at the baseline visit. *H. pylori*-positive participants (n = 19) who consented to the antibiotic treatment received antibiotics based on the standard-of-care. Subjects were given treatment with twice daily amoxicillin (1000 mg) and clarithromycin (500 mg) for 2 weeks. For the two subjects with penicillin allergy, amoxicillin was replaced by metronidazole 500 mg three times daily. Of those, 17 completed the final study visit at 6 months. Four subjects who underwent a baseline assessment and tested positive for *H.pylori* withdrew from the study before receiving any antibiotics (Fig. 1B).

### *O. formigenes* colonization status and stability over time

**Figure 2:**
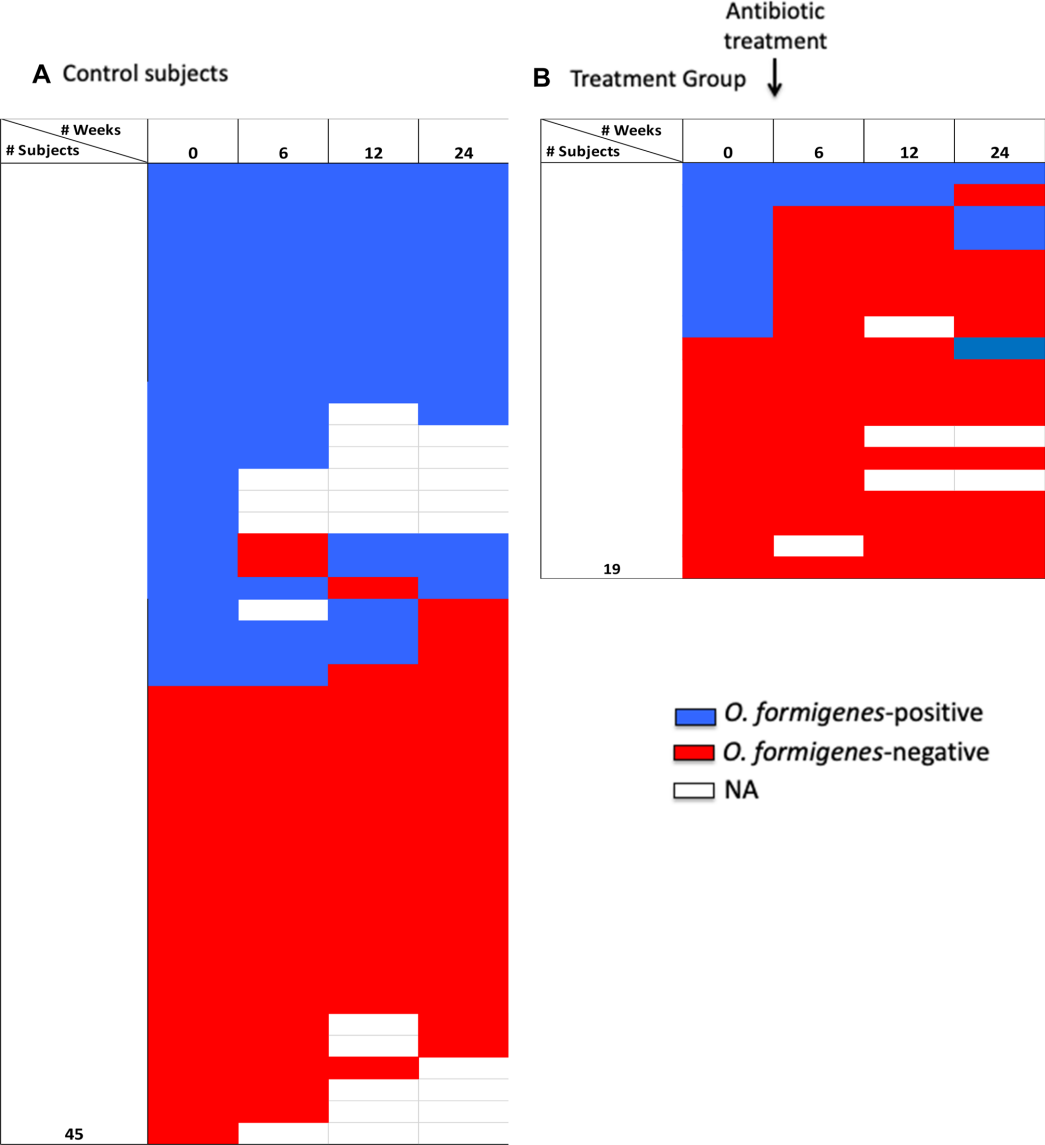
*O. formigenes* colonization status in 45 control subjects and 19 antibiotic-treated subjects over the study period. *O. formigenes*-positive samples are shown in blue, negative in red, and missing in white. Panel (**A**) Untreated control subjects. Panel (**B**) Antibiotic-treated subjects. Figure created by Excel software version 16.44.

Since there is no consensus on the best method to detect *O. formigenes* in stool samples, we developed a positivity score based on PCR, qPCR and 16S rRNA gene sequencing data at multiple time points to accurately represent *O. formigenes* positivity in the stool samples of the study subjects. Based on the *O. formigenes* positivity score, the prevalence of *O. formigenes* was 50% in the 64 participants at baseline (Figs. 1, 2), similar to prior findings in the USA^20,22,24,27^. The *O. formigenes*-positive and negative participants were similar in distributions of age, sex, ethnicity, BMI, mode of birth-delivery, *H. pylori* status, and place of birth (Table S2). We evaluated *O. formigenes* colonization status stability over time in both the antibiotic-treated participants and controls. Among the controls, of the 21 *O. formigenes*-positive subjects at baseline assessed at 6 weeks, 19 (90%) remained positive, vs. 0/20 *formigenes*-negative participants at baseline becoming positive (*p* < 0.001, by Fisher’s exact). In contrast, in the antibiotic-treated group, of the eight initially *O. formigenes*-positive participants, only two (25%) remained positive at 6 weeks vs. 19 (95%) of the 20 who were untreated (*p* < 0.001, Fisher’s exact test). Only three (37.5%) of the treated subjects remained positive at 24 weeks vs. 20 (83.3%) of 24 in the untreated controls (*p* < 0.02, Fisher’s exact test). All 11 of the baseline *O. formigenes*-negative group remained negative (Fig. 2 A, B). Thus, treatment to eradicate *H. pylori* had a strong effect, largely eliminating *O. formigenes*, confirming prior studies ^26^.

### *O. formigenes* detection by high throughput 16S rRNA gene sequencing

Our prior studies showed that *O. formigenes* abundance can vary in carriers over a range > 3-log_10_, with some individuals having minimally detectable numbers^24,28^, which intrinsically limits interpreting positivity.

**Figure 3:**
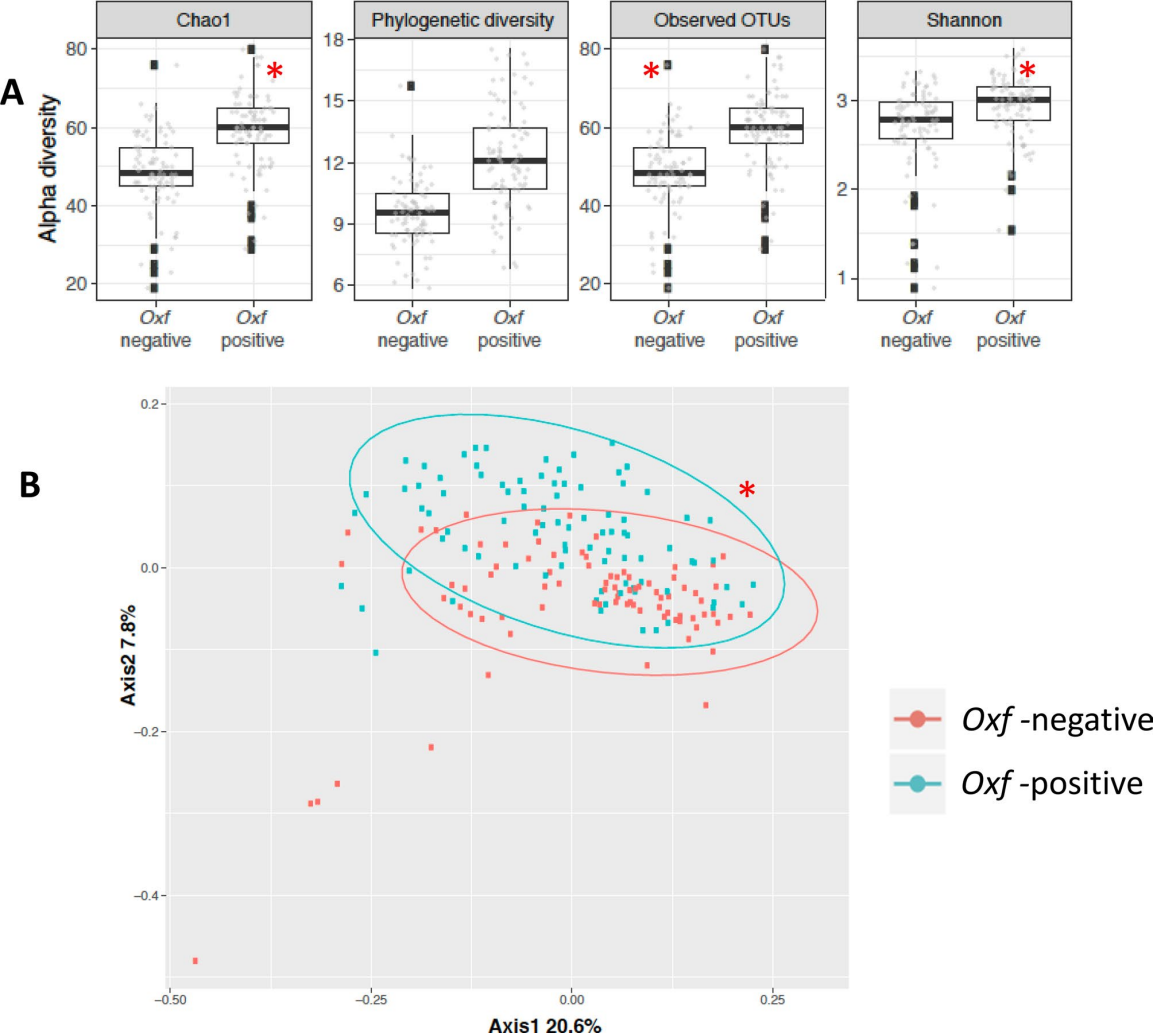
Alpha- and beta-diversity of microbiota in 173 fecal samples. from 64 study subjects, according to baseline *O. formigenes* status. Panel (**A**) Alpha-diversity measurements. Chao1, phylogenetic diversity, observed OTUs, and Shannon diversity according to *Oxf* status at baseline. Panel (**B**) Analysis of Beta-diversity, Unweighted UniFrac distances, according to *O. formigenes* status. *p < 0.05. Figure created by R software Ggplot2 package.

Using 16S rRNA gene sequencing, we detected *O. formigenes* in 10 (22%) of the 45 controls at baseline, vs. 24(53%) using the above *O. formigenes* scoring system. At follow-up, we detected *O. formigenes* in six other controls by 16S rRNA gene sequencing analysis, but positivity fluctuated for individual subjects (Fig. 3). However, across all the control subjects, the mean % relative abundance was stable over time (0.06 ± 0.0 at baseline and 0.03 ± 0.04 at week 24). At baseline in the antibiotic-treated group, *O. formigenes* was detected in 4 (21.1%) of 19 participants; after antibiotic treatment, all of these subjects remained *O. formigenes* negative at 6, 12 and 24 weeks. Two subjects who had undetectable *O. formigenes* according to the initial16S analysis (but who were positive for *O. formigenes* by the combined scoring method) had detectable *O. formigenes* 16S rRNA gene amplification after antibiotic treatment.

### Urinary parameters according to antibiotic treatment

To assess the differences in urinary electrolytes before and after *H. pylori* eradication in antibiotic-treated participants and the stability of these electrolytes in untreated controls, we collected a fasting urine sample (pre-meal urine) and then administered a standard 16-oz liquid meal totaling 700 cal (2 cans of Ensure Plus) (Table S1) and collected urine for three hours after the meal (post-meal urine) (Fig. 1A). We used a linear mixed model fitted to the data over the time interval 0–24 weeks in which the changing rates in urine parameters were compared based on baseline treatment status (control, treatment group). In both the controls and the treatment group, pre-meal UOx/Cr increased over the course of the study (*p* < 0.05), with comparable increases for the two groups. In contrast, post-meal UOx/Cr did not change over time (Table S3, Supplementary Fig. 2). Urine pH levels were unchanged in the untreated controls, as expected, but increased in the treated patients in both the pre- and post-meal comparisons (*p* = 0.04 and 0.06, respectively). Urine ammonia/Cr differed in their direction of change in controls and treated subjects in pre-meal samples but after the meal increased in both control and antibiotic-treated participants. Urine citrate/Cr was stable over time in pre-meal samples from controls and in post-meal samples from both controls and treated subjects but significantly increased in the treatment group. The ratio to creatinine of urinary sodium, phosphorus, calcium, and urea nitrogen increased pre-meal in both the control and antibiotic-treated subjects, but were unchanged in post-meal comparisons over time (Table S3). These results indicate the effects of antibiotic exposures on urinary electrolyte excretion, pertaining particularly to acid–base homeostasis.

### Microbial community structure

**Figure 4:**
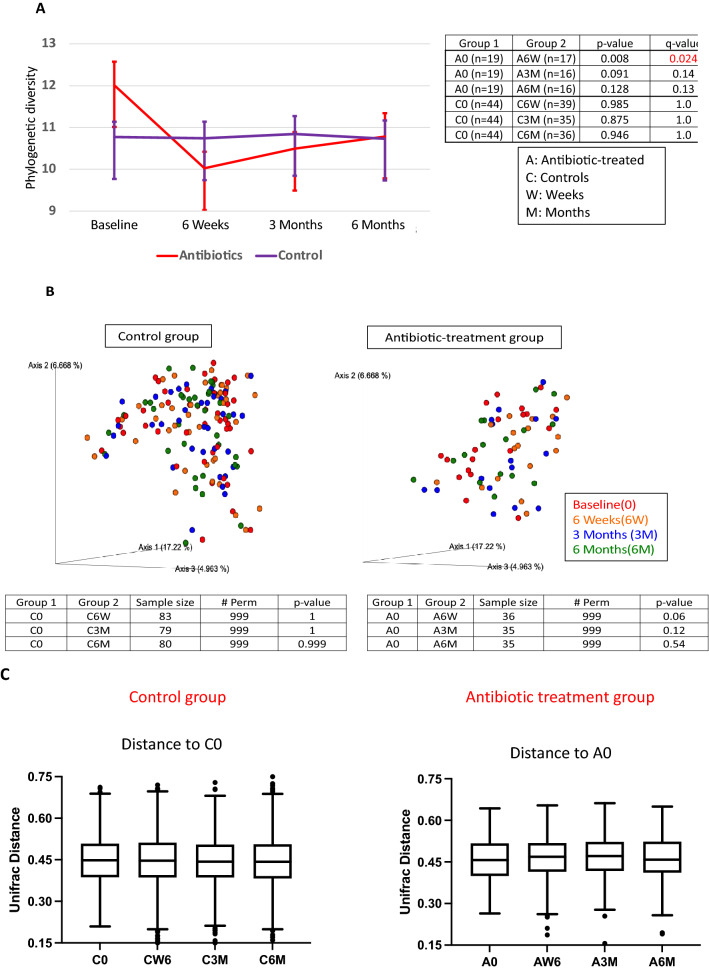
Alpha- and beta-diversity of microbiota in controls and antibiotic-treated subjects over time. Panel (**A**) Phylogenetic diversity over time in the two groups of subjects; Kruskal Wallace analysis. Panel (**B**) Unweighted UniFrac distances over time. Panel (**C**) Comparison of beta diversity between baseline and follow-up specimens in the control and antibiotic treatment groups. Box-and-whisker plots (Tukey) of unweighted UniFrac distance are shown at sequencing depth of 8,000. Comparisons were done between baseline (0) vs. follow up (W6, 3M, 6M), by Pairwise Permanova testing with 999 permutation; no differences were significant at *p* < 0.05. Panels (**A**,**B**) are created by QIIME2 software (version 2-2019.1) while Panel (**C**) was created by R software Ggplot2 package.

Alpha-diversity is used to measure the bacterial diversity within a sample, whereas Beta-diversity provides a measure of the dissimilarity between samples. First, we asked whether the microbiome differed in the study subjects according to *O. formigenes* status in control subjects or baseline assessments (before antibiotics) in the antibiotic treatment group. Fecal samples from the *O. formigenes*-positive subjects had higher α-diversity (p < 0.01, by Wilcoxon Rank test) than those from *O. formigenes-*negative subjects (Fig. 3A). After including subject as random effect in a linear mixed-effect model, we found similarly that *O. formigenes* is associated with higher α-diversity measurements (*p* < 0.01). As expected, in the untreated controls, α-diversity remained stable, but after antibiotic treatment, decreased at 6 weeks and later returned to baseline (Fig. 4A). To assess the communities for β-diversity, we examined unweighted UniFrac distances, consistent with our previous analysis of the American Gut Project data^24^, in 173 samples from 64 participants according to *O. formigenes* baseline-status; β-diversity significantly differed (*p* = 0.029 by Adonis test with 999 permutations) in the samples based on *O. formigenes* status (Fig. 3B). Similar to α-diversity, β-diversity was stable over time in untreated controls, but changed (*p* = 0.06, by pairwise Permanova testing with 999 permutation) immediately following antibiotic exposure, then returning to baseline (Fig. 4B, C). These studies provide evidence that *O. formigenes* status correlates with and clinical antibiotic regimen affects overall microbiome community characteristics.

### Association between UOx/Cr and specific bacterial taxa

To assess whether any taxa other than *O. formigenes* were positively or negatively associated with UOx/Cr, we first analyzed the controls (41 participants with 108 measurements at four time points), using a centered log-ratio (CLR) transformation to standardize relative abundances of individual microbial taxa before fitting to a linear mixed model (Table 1). The analyses showed that relative abundance of the phylum Proteobacteria was positively (Adj. *p* < 0.01) associated with urinary oxalate levels. Genus Dysgonomonas (Adj. *p* = 0.02) and species *D. gadei* (Adj. *p* < 0.01) were inversely associated with post-meal urinary oxalate level, adjusted for age, gender, ethnicity, BMI, and calcium level. Based on our model, in the antibiotic-treated subjects (Table 2), the clr transformed relative abundance of genera Dysgonomonas (Adj. *p* = 0.02), Epulopiscium (Adj. *p* = 0.02), and Providencia (Adj. *p* = 0.02) and species *Bacteroides plebeius* (Adj. *p* = 0.03) were inversely associated with pre-meal urinary oxalate level, whereas genus Lachnobacterium (Adj. p = 0.02) and species *Faecalitalea cylindroides* (Adj. *p* = 0 < 0.01) were positively associated. Family Barnesiellaceae (Adj. *p* = 0.01) and species *Streptococcus gordonii* (Adj. *p* = 0.05) were negatively associated with post-meal urinary oxalate levels, whereas order Turicibacterales (Adj. *p* = 0.01) and family Turicibacteraceae (Adj. *p* = 0.01) were positively associated. In our models, family Oxalobacteraceae was not associated with pre- and post urinary oxalate in both controls and antibiotic-treated subjects.

**Table 1:** Microbial taxa associated with pre/post-dietary urinary oxalate levels in untreated controls.

Microbial taxon	Pre-meal urinary oxalate level				Post-meal urinary oxalate level			
	Coef. Est	Std. Error	Unadj. p	Adj. p	Coef. Est	Std. Error	Unadj. p	Adj. p
Phylum proteobacteria	0.12	0.04	0.01	< 0.01	0.01	0.01	0.54	0.94
Genus dysgonomonas	− 0.04	0.05	0.51	0.94	− 0.04	0.01	< 0.01	< 0.01
Dysgonomonas gadei	− 0.02	0.05	0.67	0.96	− 0.05	0.01	< 0.01	< 0.01
Family oxalobacteraceae	0.01	0.02	0.72	0.96	0.01	0.01	0.73	0.95

The estimated regression coefficients, standard errors, and unadjusted/adjusted p-values for the effect of the relative abundance of individual microbial taxa with centered log-ratio transformation in fitted mixed effect models. Analysis adjusted for age, gender, ethnicity, BMI, and calcium level.

**Table 2:** Microbial taxa associated with pre/post-dietary urinary oxalate level in antibiotic-treated subjects.

Microbial taxon	Pre-meal urinary oxalate level				Post-meal urinary oxalate level			
	Coef. Est	Std. Error	Unadj. p	Adj. p	Coef. Est	Std. Error	Unadj. p	Adj. p
Order turicibacterales	0.05	0.02	0.05	0.31	0.02	0.01	< 0.01	0.01
Family barnesiellaceae	− 0.10	0.04	0.04	0.50	− 0.04	0.01	< 0.01	0.01
Family turicibacteraceae	0.04	0.02	0.08	0.60	0.02	0.01	< 0.01	0.01
Genus dysgonomonas	− 0.40	0.11	< 0.01	0.02	− 0.04	0.02	0.03	0.25
Genus epulopiscium	− 0.43	0.12	< 0.01	0.02	− 0.05	0.02	0.03	0.25
Genus lachnobacterium	0.08	0.01	< 0.01	0.02	0.01	< 0.01	0.03	0.25
Genus providencia	− 0.43	0.12	< 0.01	0.02	− 0.05	0.02	0.02	0.25
Bacteroides plebeius	− 0.12	0.03	< 0.01	0.03	− 0.01	< 0.01	0.21	0.72
Streptococcus gordonii	− 0.07	0.06	0.22	0.78	− 0.04	0.01	< 0.01	0.05
Faecalitalea cylindroides	0.24	0.05	< 0.01	< 0.01	0.02	0.02	0.33	0.77
Family oxalobacteraceae	0.07	0.06	0.24	0.82	-0.01	0.01	0.83	0.97

The estimated regression coefficients, standard errors, and unadjusted/adjusted p-values for the effect of the relative abundance of individual microbial taxa with centered log-ratio transformation in fitted mixed effect models. Analysis adjusted for age, gender, ethnicity, BMI, and calcium level.

## Discussion

In this study, we confirmed that antibiotic treatment has a strong effect on *O. formigenes* status^26,29^. Although most antibiotic-treated participants had durable suppression, lasting > 24 weeks, *O. formigenes* persisted in some, suggesting either heterogeneity in antibiotic resistance of *O. formigenes* strains or inability of the active agent(s) to achieve sufficient colonic concentrations. Since *O. formigenes* colonization has been associated with lower oxaluria^30^, our observation of treatment effects provides one explanation for why antibiotic treatments have been associated with a higher kidney stone incidence^12,31^.

This study highlights the difficulty in assessing patients’ *O. formigenes* status due to the wide range in colonization density that may reflect both diet and competing organisms. This variation confirms our prior work^24,28^, which provided the rationale for using a formula based on multiple tests to detect colonization. We therefore developed a positivity score based on PCR, qPCR and 16S rRNA gene sequencing data at multiple time points to more accurately represent *O. formigenes* positivity in our stool samples. This biological variation is likely also in part responsible for the continued uncertainty about whether *O. formigenes* status is associated with nephrolithiasis or not. Our results may more accurately represent colonization of individual subjects than previously since prior studies used a single assessment types done cross-sectionally, which can underestimate colonization prevalence. Giving subjects an oxalate-rich diet that selects for *O. formigenes* growth may enhance detection in future cross-sectional analyses. Finding that 16S rRNA gene sequencing has low sensitivity for *O. formigenes* detection, due to its often low abundance, may be useful in future studies where colonization needs to be confirmed using more sensitive methods.

The standard meal used in this study did not contain a substantial oxalate load because the original trial was not designed to measure our primary outcome. Therefore, urinary oxalate changes might not reach those observed with moderate-high oxalate diets that healthy individuals typically consume. Differences in UOx/Cr (indicated by the standard deviations in Supplementary Fig. 2) emphasize the need for larger trials with high oxalate, low calcium, and controlled diets. However, urinary pH increased after antibiotic treatment but not in controls, indicating possible effects beyond oxalate metabolism affecting acid–base homeostasis. The recent associations between increased stone risk and antibiotic courses did not include urinary chemistry data in one report^12^, and in the second, the temporal relationship between antibiotic use and urine collection was not standardized^13^. Our findings provide potential mechanisms for the linkage between prior antibiotic use and nephrolithiasis risk. Higher urinary pH is associated with increased risk for calcium phosphate stones^32^.

Our community structure findings indicate that *O. formigenes* presence is a marker for a richer microbiome (consistent with prior studies^21,24^) and its loss is a marker of important antibiotic-induced microbiome alterations. That no significant recolonization was detected by 6 months, while the overall diversity recovered, is consistent with persistent microbiome disturbance.

We identified a group of bacteria positively or negatively correlated with urinary oxalate level, consistent with the hypothesis that oxalate degradation in the gut is affected by the putative *oxalobiome* that facilitates oxalate degradation^8^. This study provides the first analysis of such associations in healthy subjects to potentially identify a microbiome associated with differential potential for stone formation. Since our findings indicate taxa with abundance associated with lower urinary oxalate levels, these could be protective against hyperoxaluria; species from genus Providencia are known to be oxalate-degraders^33^. Similarly, we identified bacteria associated with higher urinary oxalate levels whose presence could potentially increase kidney stone risk^9^. Proteobacteria were associated with higher urine oxalate in our study and were enriched in stone formers in two other studies^9,11^.

The lack of an association of *O. formigenes* colonization with lower urinary oxalate excretion could reflect its very low abundance in subjects without dietary oxalate enrichment and insensitive detection using 16S rRNA gene sequencing. Future studies with deeper coverage will facilitate identifying low-abundance bacterial taxa.

Our study was limited by the small sample size for the antibiotic-treated participants and lack of information about baseline diets. The standard meal did not contain oxalate, and the 3-h post-meal urine collection did not permit study of less acute colonic bacterial actions on dietary components.

In conclusion, our study found substantial antibiotic effects on *O. formigenes* colonization, its relationship with other taxa and overall microbiome community structure, and with several urinary parameters, and identified a consortium of bacterial taxa associated with urinary oxalate levels. In the future, understanding metabolic activities of the intestinal taxa may be useful for primary kidney stone prevention.

## Materials and methods

### Recruitment and subject enrollment

Participants were recruited for the ESSAY study (Eradication Study in Stable Adults/Youths) evaluating the effect of the standard-of-care practice of *H. pylori* eradication on metabolic profile and anthropometric measures of healthy adults. Participants were identified from the Bellevue Hospital primary care clinic, and community. Healthy young adults who were 18–40 years old were screened by research coordinators for eligibility criteria and then signed an informed consent if meeting those criteria (Fig. 1B). In total, 139 participants were screened for participation. Of the 87 participants who met the eligibility requirements and provided informed consent, 69 completed the baseline visit while 18 were lost to follow-up (Fig. 1B). The clinical study was conducted between April 2012 and July 2016. We excluded participants with diabetes, hyper- or hypothyroidism, prior gastric or bariatric surgery, prior *H. pylori* treatment, steroid or other immunomodulatory drug use within 4 weeks of the first visit, and antibiotic use within the prior 6 months. Participants completed baseline questionnaires to provide their demographic information, medical history, and current medication use. The study was approved by the Institutional Review Board (IRB) at NYU Langone Health. All research was performed in accordance with the Declaration of Helsinki and our local IRB guidelines. Informed consent was obtained from all participants.

### Determination of *H. pylori* status at baseline and at follow-up and antibiotics regimen

Subjects underwent a non-radioactive ^13^C Urea Breath Test (Meretek Diagnostics, New York NY) to determine *H.pylori* status. Subjects (n = 23) who tested *H. pylori-*positive were offered treatment with a 14-day twice-daily regimen [amoxicillin 1000 mg, clarithromycin 500 mg, and proton pump inhibitor (PPI; omeprazole 20 mg, rabeprazole or esomeprazole 40 mg)], per the then-current standard of care^34^. In total, 19 subjects received a course of antibiotics, including three who received a second antibiotic course because they failed eradication with the first course, and four other subjects withdrew from the study before receiving antibiotics (Fig. 1). The 46 subjects who were *H.pylori* negative at baseline did not receive antibiotics and were followed serially as controls.

### Study time points and assessments

Subjects fasted overnight at home and then underwent basic assessment at the NYU Clinical and Translational Science Institute (CTSI) at Bellevue Hospital at the baseline and 6, 12, and 24-week timepoints and height and weight obtained. Stool samples were collected and placed immediately on ice at home and brought to the center within 24 h, then immediately frozen at − 80° C. No preservatives were used and the kits were prepared in-house. In total, we obtained fecal samples from 65 subjects at baseline (Fig. 1).

### Test meal and urine collections

To assess the differences in urinary electrolytes before and after *H. pylori* eradication, we collected a fasting urine sample and then administered a standard 16-oz liquid meal totaling 700 cal (2 cans of Ensure Plus) (Table S1). Urine was collected for three hours after the meal. In total, we collected urine samples (pre and post) from 32 controls and 14 treated subjects, (Fig. 1). Urine samples were collected at the CTSI during the study visit.

### DNA isolation and 16S rRNA gene sequencing

The fecal DNA was extracted using the PowerSoil-htp 96-Well DNA Isolation Kit (MoBio, Carlsbad CA, USA), following the manufacturer’s instructions which includes mechanical and chemical lysis steps. The V4 region of bacterial 16S rRNA genes was amplified in triplicate reactions using barcoded fusion primers 515F/806R, which amplifies bacterial and archaeal 16S genes^35,36^. The DNA concentration of the V4 amplicons for each sample was measured using the Quant-iT PicoGreen dsDNA assay kit (Life Technologies, Eugene OR, USA), and samples pooled in equal quantities. These pools were treated with the Qiaquick PCR purification kit (Qiagen) to remove primers, quantified using the high-sensitivity dsDNA assay kit and the Qubit 2.0 Fluorometer (Life Technologies, Eugene OR, USA) and then combined at equal concentrations to form the sequencing library. The ~ 254 bp V4 region was sequenced using the Ilumina MiSeq 2 × 150 bp platform at the New York University Langone Medical Center (NYULMC) Genome Technology Center. We included negative controls during the DNA extraction and library prep that were also sequenced**.**

### *Oxalobacter formigenes* quantitative PCR

qPCR was used to quantitate the number of copies of *O. formigenes* oxc using the *O. formigenes* specific primers (Forward 5′-GTG-TTG-TCG-GCA-TTC-CTA-TC-3′, Reverse 5′-TTG-GGA-AGC-AGT-TGG-TGG-3′). qPCR was performed using the LightCycler 480 SYBR Green I Master Mix, primers targeting *oxc*, in the LightCycler 480 system (Roche, Pleasanton CA). Melting peak analysis was performed from 65 to 95 °C to confirm amplicon specificity. A positive result was defined by amplification greater than 1.0E2, with melting peak between 86–87 °C.

### 16S rRNA gene sequencing analysis

Quantitative insights for microbial ecology (QIIME, Version Qiime 2–2019.1) was used for quality filtering and downstream analysis for α-diversity, β-diversity, and compositional analysis^37^. Sequences were filtered for quality trimmed, de-noised, and merged, and then the chimeric sequences were removed using the DADA2 plugin (version 1.6.0.) using default parameters to generate the feature table containing amplicon sequence variants (ASV)^38^. QIIME 2 was used to generate a rooted phylogenetic tree to be used for phylogenetic analyses. Phylogenetic diversity, observed features number, Chao1, and Shannon indices were used as α-diversity measures of the intra-individual diversity. The inter-individual (β-) diversity was computed as unweighted UniFrac distances and differences in β-diversity were visualized with principal coordinates analysis (PCoA) plots. Taxonomy was assigned using GreenGenes 13_8 for reference.

### Sample diversity analyses

Intra-sample α-diversity was calculated using QIIME2, using phylogenetic diversity, observed OTU number, Chao1, and Shannon indices at rarefaction depths of 8000 sequences/sample. Beta-diversity was assessed using the unweighted UniFrac distance metric^24^.

### *Oxalobacter formigenes* status determination

We tested each fecal sample for the presence of *O. formigenes* using PCR in duplicate, qPCR, and 16S rRNA gene sequencing. Based on the known several log-fold biological variations in *O. formigenes* abundance in human fecal specimens^24,28^, given the ascertainment variability using different methods, we developed a positivity score based on the 42 control subjects who did not receive antibiotics and who were tested at baseline and at least once in follow-up. The maximum number of assessments for each test subject was 16 if the participant was tested at all 4 time points (baseline, weeks 6, 12, 24) in all four assays; and a minimum of 8 if only tested at two time points. For the 42 control participants, the mean ± SD number of determinations was 14.7 ± 2.6. We computed the positivity score by dividing the number of positive assessments by the total number of assessments for that subject. Since the results were bimodal, as expected (Supplementary Fig. 1) we assigned baseline *O. formigenes* status as negative with score ≤ 0.2 and positive with score ≥ 0.4. Only one subject had a score of 0.3, which we considered indeterminate and we removed this participant from further analysis (Supplementary Fig. 1). Similarly, per sample positivity score was used to assign *O. formigenes* status at different time points with the criteria: negative with score ≤ 0.2 and positive with score ≥ 0.4.

### Urine testing

Urine aliquots were mixed with either HCl (for oxalate measurements) or with thymol (for all other assessments) and then stored at − 80 °C. Pre-meal (fasting) and the 3-h post-meal urinary samples were analyzed by Litholink Corporation (Chicago IL)^39,40^. In each urine sample, we measured calcium, chloride, creatinine, magnesium, sodium, potassium, phosphate, and ammonium concentrations by standard techniques by Beckman Synchron AU680 (Beckman Instruments, Brea CA), as described^40^; pH was measured by glass electrode. Oxalate was measured by enzyme assay using oxalate oxidase (Trinity Biotech, Bray, Ireland). Citrate was measured by enzyme assay using citrate lyase (Mannheim Bohringer, Mannheim, Germany). All urine parameters except pH were normalized by dividing by creatinine concentration to account for hydration status.

### Longitudinal analysis of urine parameters

A linear mixed-effects model was fitted for urine parameters over the time interval 0–24 weeks. By involving measurement time as the fixed effect and subject as the random effect, we evaluated the changing rates during this period. Other covariates age, sex, and body mass index (BMI) were also included for adjustment. The *p*-value < 0.05 was considered significant. Analysis was conducted with R statistical software using the lmerTest package. Satterthwaite approximation was employed to get p-value.

### Association between Uox/Cr and bacterial taxa

Linear mixed-effects models were fitted to ascertain which microbial taxa are associated with pre- or post-meal urinary oxalate level (or other urinary parameters), respectively. In all models, the microbial factor of interest, α-diversity for the entire bacterial community, or the centered log-ratio transformed relative abundance of each taxon at a given taxonomic rank (phyla, classes, orders, families, genera, and species), and measurement time were included as fixed effects. The intercept and slope of the linear time trend for each subject were included as random effects. Covariates age, gender, ethnicity, BMI, and calcium level were also involved as fixed effects for adjustment. The Benjamini-Hochberg (BH) procedure was applied for multiple testing correction within each taxonomic level. The p-value or adjusted p-value < 0.05 was considered significant. Analysis was conducted with R statistical software using the lmerTest package. Satterthwaite approximation was employed to get p-value. We included data for the participants in (i) the control group, we analyzed a total of 108 pairs of microbial measurements and urinary oxalate level (pre-/post- dietary) at baseline, 6, 12 and 24 weeks; and (ii), in the antibiotic-treatment group, we analyzed a total of 51 pairs of microbial measurements and urinary oxalate level (pre-/post- dietary) at 6, 12, and 24 weeks. A total of 233 taxa were examined at all taxonomic levels (phylum to species). We Included taxa with mean relative abundances among subjects > 10^–5^. For the adjusted p-values, the Benjamini–Hochberg procedure was applied for each taxonomic level. Unclassified microbial taxa were not included in the analysis. Analysis was conducted with R statistical software using the lme4 package. Unclassified microbial taxa were not included in the analyses.

## Supplementary Information

Supplementary Information.
