# Supplementary material for: Effect of antibiotic treatment on Oxalobacter formigenes colonization of the gut microbiome and urinary oxalate excretion
Source: Sci Rep. 2021 Aug 12;11:16428. doi: 10.1038/s41598-021-95992-7 (PMC8361114; doi:10.1038/s41598-021-95992-7)
Supplement: Supplementary file 1 — Supplementary Information. [file 41598_2021_95992_MOESM1_ESM.docx]

Effect of antibiotic treatment on *Oxalobacter formigenes* colonization of the gut microbiome and urinary oxalate excretion.

**Lama Nazzal**, Fritz Francois, Nora Henderson, Menghan Liu, Huilin Li, Hyunwook Koh, Chan Wang, Zhan Gao, Guillermo Perez Perez, John R. Asplin, David S Goldfarb, Martin J Blaser

**Legends to Supplemental Figures**

**Supplemental Figure 1: Histogram of individual *O. formigenes* positivity scores in 39 non-antibiotic controls.** Scores were computed by dividing the number of positive assessments using qPCR, 16S rRNA gene sequencing, and PCR in duplicate, by the total number of assessments for *Oxf* across 4 time points (total of 16 assessments/subject). *Oxf*+ was defined as values > 0.4, *Oxf* - as <0.20, and the one subject with a score of 0.3 was considered as Indeterminate. GraphPad Prism software was used to create the figure.

**Supplemental Figure 2: Pre- and Post-meal Ox/Cr (mg/g) in 32 untreated controls** **and 14 antibiotic-treated subjects. Left Panels**: Untreated controls. **Right Panels**: Treated subjects. **Top panels**: Baseline assessments. **Bottom Panels:** Samples obtained at week 24. Data presented with mean and standard deviation. GraphPad Prism software was used to create the figure.

| Table S1 : Nutrients present in Ensure* | | |
| --- | --- | --- |
| **Component** | **Present**  **in Ensure** | **Recommended**  **Daily Value (%)** |
| Oxalate | No | 0 |
| Vitamin C | Yes | 50 |
| Calcium | Yes | 30 |
| Chloride | Yes | 8 |
| Potassium | Yes | 11 |
| Magnesium | Yes | 25 |
| Sodium | Yes | 8 |
| Phosphorus | Yes | 25 |
| Protein | Yes | 18 |

*Ensure, nutritional supplement manufactured by Abbott Laboratories.

| Table S2: Demographics of 64 study subjects by *O. formigenes* status at baseline | | | |
| --- | --- | --- | --- |
|  | *O. formigenes-*     positive (n=32) | *O. formigenes-*       negative (n=32) | P value |
| Age (Years) | 26.6 + 6.2 | 24.0 + 5.1 | 0.1 |
| Sex (Male/Female) | 13/19 | 10/22 | 0.6 |
| Ethnicity (White/AA/Asian/Hispanic/Other) | 11/5/9/6/1 | 11/3/12/5/1 | 0.3 |
| BMI (kg/m^2^) | 24.5 + 4.8 | 23.1 + 3.5 | 0.2 |
| Mode of delivery (Vaginal/C-section) | 21/8 | 19/4 | 0.4 |
| Place of birth (US/Non-US) | 13/19 | 16/16 | 0.6 |
| *H. pylori* status (positive/negative) | 8/24 | 11/21 | 0.6 |

| Table S3: Urinary electrolytes in controls and treated subjects | | | | | | | | |
| --- | --- | --- | --- | --- | --- | --- | --- | --- |
|  | **Controls** | | | | **Treated** | | | |
| Electrolyte | Pre–time 0  (n=32) | Pre-Time 24 (n=23) | Post–time 0 (n=32) | Post-Time 24 (n=14) | Pre–time 0  (n=14) | Pre-Time 24 (n=11) | Post–time 0 (n=14) | Post-Time 24 (n=11) |
| pH | 6.3 + 0.7 | 6.2 + 0.6 | 6.6 + 0.6 | 6.5 + 0.6 | 6.2 + 0.5 | 6.6 + 0.6* | 6.6 + 0.5 | 6.8 + 0.4* |
| Ox/Cr | 15.0 + 5.1 | 18.5 + 10.7* | 15.4 + 4.7 | 16.6 + 10.5 | 13.0 + 5.1 | 18.8 + 10.3* | 16.2 + 4.2 | 16.6 +9.0 |
| Amm/Cr | 1.9 + 0.7 | 2.6 + 1.2^┼┼^ | 2.6 + 0.7 | 3.6 + 1.8* | 2.3 + 0.5^┼^ | 2.00 + 0.6^┼┼^ | 2.3 +0.9 | 2.6 + 1.0* |
| Ca/Cr | 3.1 + 2.5 | 5.2 + 3.6* | 12.1 + 6.3 | 14.6 + 7.9* | 3.3 + 2.1 | 3.5 + 2.1* | 11.2 + 5.8 | 11.3 + 6.1 |
| Cit/Cr | 2.7 + 2.0 | 2.7 + 1.8 | 4.4 + 2.4 | 4.4 + 2.2 | 2.1 + 0.9 | 2.9 + 1.8* | 3.9 + 2.0 | 4.2 + 2.4 |
| Cl/Cr | 6.2 + 3.0 | 9.3 + 4.6* | 8.8 + 3.5 | 10.7 + 4.2 | 9.0 + 4.4 | 9.4 + 5.6* | 10.8 + 3.8 | 11.7 + 4.0 |
| K/Cr | 3.5 + 2.0 | 4.2 + 1.8 | 7.3 + 2.9 | 8.8 + 3.7 | 3.3 + 1.5 | 4.6 + 2.3 | 2.8 + 1.3^┼^ | 3.8 + 1.3 |
| Mg/Cr | 3.2 +1.4 | 4.0 + 1.6 | 7.8 + 3.4 | 8.5 + 3.4 | 3.4 + 1.0 | 3.7 + 1.8 | 7.7 + 3.2 | 8.5 + 4.1 |
| Na/Cr | 4.5 + 2.2 | 7.1 + 3.8* | 7.3 + 2.9 | 8.8 + 3.7 | 7.0 + 3.2 | 7.4 +3.8* | 9.6 + 3.9 | 10.7 + 3.7 |
| Phos/Cr | 2.8 + 1.1 | 3.7 + 1.5* | 3.6 + 1.6 | 3.9 + 2.0 | 2.9 + 1.7 | 3.1 +1.2* | 2.9 + 1.3 | 3.8 + 1.8 |
| UN/Cr | 4.6 + 1.6 | 5.6 +1.8* | 6.4 + 1.9 | 7.2 + 2.0 | 5.4 + 1.4 | 5.6 + 1.4* | 6.6 + 1.0 | 6.7 + 1.4 |

Cr: creatinine (mg/dL), Ox: oxalate (mg/dL), Amm: ammonium (mmol/L)Ca: calcium (mg/dL), Cit: citrate (mg/dL), Cl: chloride (mmol/L), K: potassium (mmol/dL), Mg: magnesium (mg/dL), Na: sodium (mmol/L), Phos: phosphorus (mg/dL), UN: Urea Nitrogen (mg/dL)

+ values significantly different at baseline (yellow)

++ significantly increased in controls and decreased in treated subjects (green)

*p< 0.05, comparison between time 0 and 24, significant increase shown in blue

*
